# Development of Methodology for Disability-Adjusted Life Years (DALYs) Calculation Based on Real-Life Data

**DOI:** 10.1371/journal.pone.0074294

**Published:** 2013-09-20

**Authors:** Ellen A. Struijk, Anne M. May, Joline W. J. Beulens, G. Ardine de Wit, Jolanda M. A. Boer, N. Charlotte Onland-Moret, Yvonne T. van der Schouw, H. Bas Bueno-de-Mesquita, Jeljer Hoekstra, Petra H. M. Peeters

**Affiliations:** 1 Julius Center for Health Sciences and Primary Care, University Medical Center Utrecht, Utrecht, The Netherlands; 2 National Institute for Public Health and the Environment (RIVM), Bilthoven, The Netherlands; 3 Department of Gastroenterology and Hepatology, University Medical Centre Utrecht, Utrecht, The Netherlands; Cardiff University, United Kingdom

## Abstract

**Background:**

Disability-Adjusted Life Years (DALYs) have the advantage that effects on total health instead of on a specific disease incidence or mortality can be estimated. Our aim was to address several methodological points related to the computation of DALYs at an individual level in a follow-up study.

**Methods:**

DALYs were computed for 33,507 men and women aged 20–70 years when participating in the EPIC-NL study in 1993–7. DALYs are the sum of the Years Lost due to Disability (YLD) and the Years of Life Lost (YLL) due to premature mortality. Premature mortality was defined as death before the estimated date of individual Life Expectancy (LE). Different methods to compute LE were compared as well as the effect of different follow-up periods using a two-part model estimating the effect of smoking status on health as an example.

**Results:**

During a mean follow-up of 12.4 years, there were 69,245 DALYs due to years lived with a disease or premature death. Current-smokers had lost 1.28 healthy years of their life (1.28 DALYs 95%CI 1.10; 1.46) compared to never-smokers. The outcome varied depending on the method used for estimating LE, completeness of disease and mortality ascertainment and notably the percentage of extinction (duration of follow-up) of the cohort.

**Conclusion:**

We conclude that the use of DALYs in a cohort study is an appropriate way to assess total disease burden in relation to a determinant. The outcome is sensitive to the LE calculation method and the follow-up duration of the cohort.

## Introduction

In the last decades improved survival of patients with several chronic diseases has led to an increase in life expectancy. As a consequence, the prevalence of chronic diseases, such as cardiovascular diseases and cancer has grown [1], [2]. Therefore, assessing disease burden by either morbidity rates or mortality rates for individual diseases may result in different conclusions. Summary health measures, combining morbidity and mortality, may offer better insight in the true burden of chronic diseases.

The World Health Organization and the World Bank developed such a composite measure called Disability-Adjusted Life Years (DALYs) [3], [4]. The primary reason was to create a single estimate that aggregates the burden of disease on population level in Global Burden of Disease studies [5], [6]. Additionally DALYs were used to define how many DALYs were attributable to several lifestyle factors [7]. DALYs are also used in economic evaluations and risk-benefit assessments [5], [8], [9].

DALYs are the sum of the Years Lost due to Disability (YLD) and the Years of Life Lost (YLL) due to premature mortality [10]. The YLD in a population are calculated by the number of years persons live with a disability multiplied by a disability weight reflecting the severity of the disability. This weight varies between 0 (no burden) and 1 (mortality). The YLL are computed as the number of years that death occurs earlier than expected. The expected number of life years is often set equal to the statistical life expectancy at birth or to the remaining years that a person of a certain age may be expected to live on average. One DALY represents the loss of one year in full health.

The burden of risk factors such as smoking or alcohol is traditionally expressed in terms of relative risks or attributable risk fractions for a specific disease. The use of summary measures has the advantage that the association with total instead of a specific disease burden is estimated. Therefore, risk factors with small effects on several diseases might be more easily identified as important for public health. Summary measures will also help estimating the overall effect of risk factors that may have opposing effects on different diseases.

So far, DALYs were mainly calculated on a population level based on statistical population data on disease incidence and mortality, but not prospectively associated with risk factors. Real-life follow-up data enables us using observed instead of modeled data with regard to risk factors, incidence and mortality event rates and confounding factors. This allows us to investigate relations of detailed risk factor information with overall disease burden. We introduce the method of DALY computation based on individual data from an ongoing follow-up study to estimate the association between risk factors and total disease burden using DALYs. Our aim is to address some methodological issues related to the computation of DALYs in real-life follow-up data from a left- and right- truncated cohort. We use the estimated number of DALYs for smoking as an example.

## Methods

We calculated DALYs for each individual in the cohort based on the YLD and YLL. Important parameters for the calculation of YLD and YLL are: disability weight of a disease, time of onset (duration) of a disease, time of death and expected time of death. The details of the calculation method including the ascertainment of the disease endpoints, calculation of the life expectancy and the analysis in relation to smoking status are presented.

The DALYs were computed for 33,507 participants of the Dutch part of the European Prospective Investigation into Cancer and Nutrition (EPIC-NL) study [11]. All participants provided written informed consent before study inclusion. The study complies with the Declaration of Helsinki and was approved by the institutional board of the University Medical Center Utrecht (Prospect) and the Medical Ethical Committee of TNO Nutrition and Food Research (MORGEN). More details about the EPIC-NL cohort are available as supporting information (EPIC-NL description S1).

Participants were followed for mortality and morbidity through linkage with several registries. Information on vital status and the date of death was obtained through linkage with municipal registries. The cause of death was obtained from Statistics Netherlands. Information on cancer occurrence was obtained from the National Cancer Registry. Other disease occurrence (Coronary Heart Disease, Cerebrovascular Accident, Diabetes Mellitus, Chronic Obstructive Pulmonary Disease, Asthma, Parkinson’s disease, Rheumatoid Arthritis, Osteoarthritis, and Inflammatory Bowel Disease) was obtained from the national hospital discharge diagnosis database from the Dutch National Medical Registry and self-report. Additional information is available as supporting information (EPIC-NL description S1). Follow-up was complete until 31 December 2007.

### Years Lost due to Disability (YLD)

**Figure 1:**
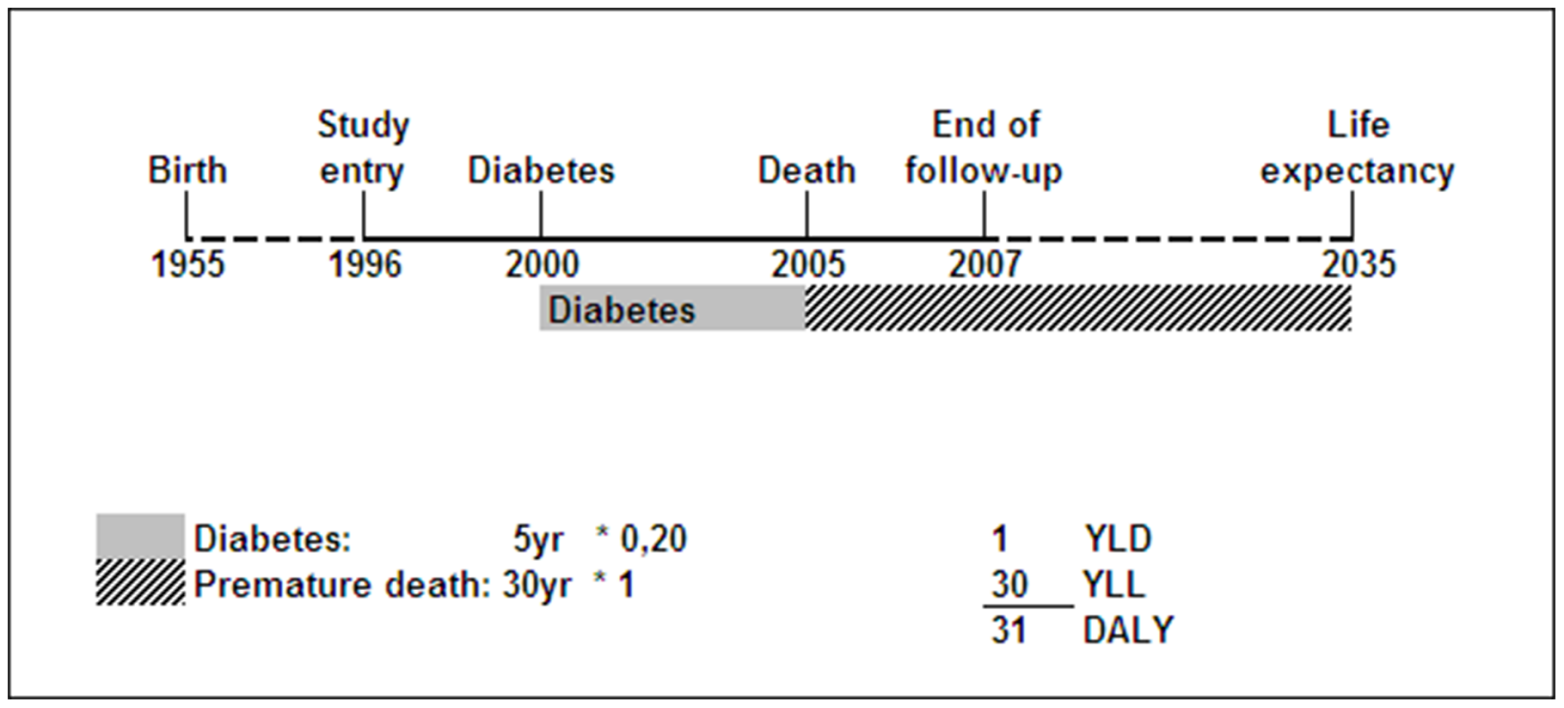
Example of DALY calculation for a deceased participant. Abbreviations: YLD, Years Lost due to Disability; YLL, Years of Life Lost; DALY, Disability-Adjusted Life Year. The disability weights are 0.20 for Diabetes and 1 for death.

**Figure 2:**
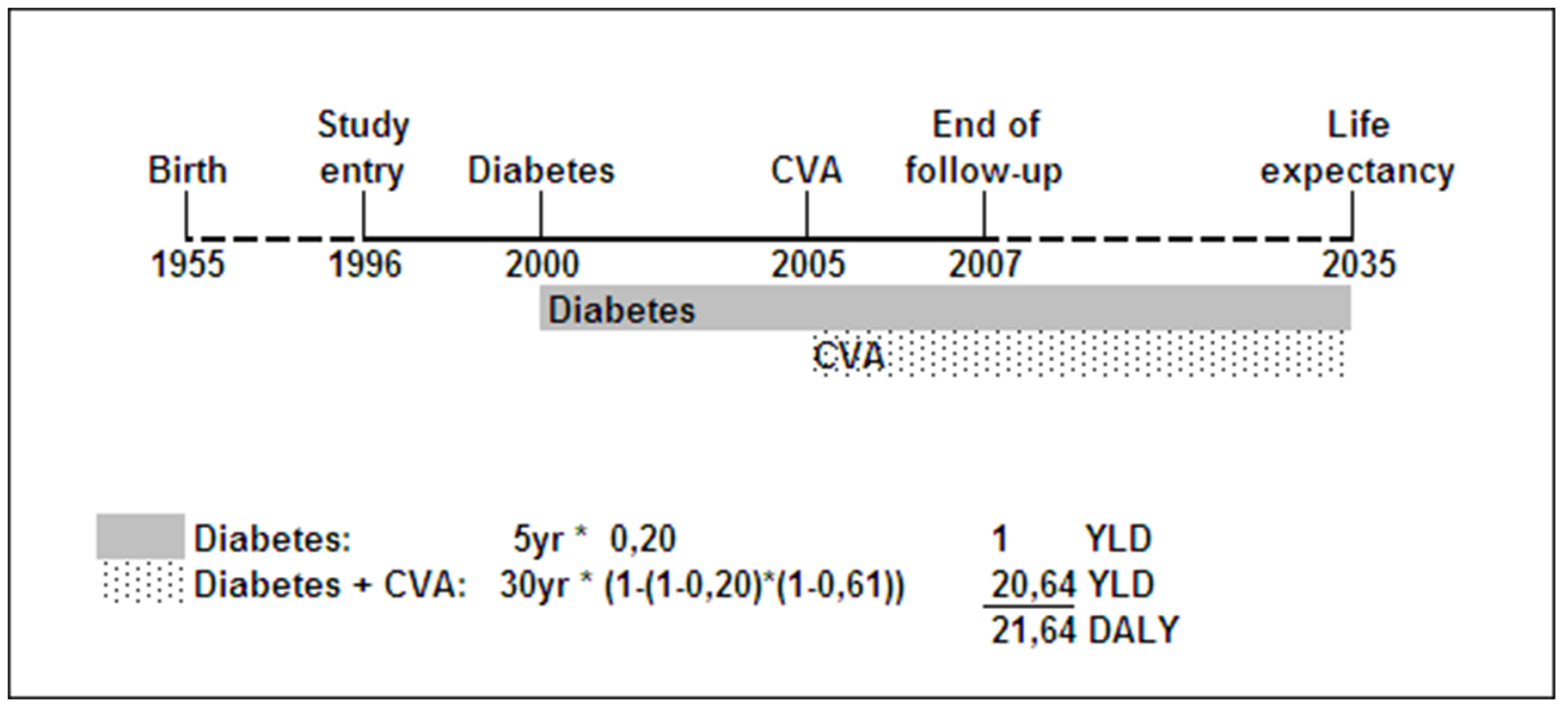
Example of DALY calculation for a participant still alive at truncation of follow-up. Abbreviations: CVA, Cerebrovascular Accident; YLD, Years Lost due to Disability; YLL, Years of Life Lost; DALY, Disability-Adjusted Life Year. The disability weights are as follows; 0.20 for Diabetes, 0.61 for CVA and 1 for death.

YLD are the number of years lived with a disability multiplied with a disability weight reflecting the severity of the disability. These weights were derived from the Dutch Disability Weight study and can range from 0 (no loss) to 1 (death) [10], [12]. For example, Diabetes Mellitus has a disability weight of 0.20. One year lived with Diabetes Mellitus is thus equal to a loss of 0.20 year [10] (2.4 months) of good health; and 5 years lived with Diabetes Mellitus equals a loss of 1 year of healthy life. Years lived with disability were calculated from date of diagnosis of a disease until date of death (figure 1) or, if a person is still alive at the truncation of follow-up (December 2007) or lost to follow-up before truncation date (for 1.9% of the total cohort), until the end of estimated life expectancy (figure 2).

For some types of cancer that were registered within EPIC-NL, disability weights were not available from the Dutch Disability Weight study [12]. The disability weights for thyroid-, brain- and bone cancer were based on weights that were derived within the Australian burden of disease study [13]. Other cancer types were assigned the same disability weight as a type of cancer with assumed comparable severity by the authors including a cancer epidemiologist and a quality of life expert (see Table S1).

Some EPIC-NL participants (n = 1,080) developed multiple diseases (comorbidity). No disability weights are available for years lived with multiple diseases. We used a multiplicative method to estimate weights for comorbid conditions which is in line with the method used in the Global Burden of Disease calculation [14], [15].

### Years of Life Lost (YLL)

YLL are defined as the number of years that death occurred earlier than the age the person was expected to die if he/she had not suffered from the disorder causing death. For example, an individual with a life expectancy until 2035 who dies in 2005 loses 30 years of life due to premature death (figure 1).

The life expectancy of a person can be calculated in several ways. Basically, the estimated life expectancy depends on two factors, i.e. attained age and calendar year in which this age is attained. Younger people have longer life expectancies than elderly, but life expectancy also changed over past calendar years, i.e. life expectancy is higher for later calendar years. Both, age and reference calendar year may be assumed to be either constant or variable, i.e. the same for each individual or depending on the individual. Combining age (constant or variable) and reference year (constant or variable) resulting in four combinations as basis for the life expectancy calculation. We chose a logical constant and variable value for age and for reference year.

Method 1: life expectancy estimated at the calendar year of birth (so: age is constant ( = 0), and the reference calendar year is variable, i.e. the individuals birth year).
Method 2: life expectancy estimated at the average age of the population at start of the observation (age is constant (49), and reference calendar year is constant (study entry, 1995). This results in a fixed life expectancy of 77.33 years and 82.51 years for men and women respectively.
Method 3: life expectancy estimated at attained age (of death or end of follow-up if alive) and the reference calendar year is year of study entry (age is variable, and reference calendar year is constant, 1995).
Method 4: life expectancy estimated at attained age (of death or end of follow-up if alive) and the reference calendar year of death or end of follow-up if alive (age is variable and reference calendar year is variable i.e. for deceased individuals calendar year of death and for alive subjects 2007).

We compared these four methods using gender specific mortality rates from Statistics Netherlands [16]. Life expectancies for birth year were only available from 1950 onwards. Therefore, participants born before 1950 were assigned the same life expectancy as participants born in 1950 (method 1). Participants who are still alive beyond their estimated life expectancy will not obtain DALYs for the period they lived longer than their estimated life expectancy (method 1 and 2).

### Statistical Analysis

The use of DALYs as a continuous outcome of disease burden is illustrated for current- and former-smokers compared to never-smokers (reference) as defined at baseline. Due to the large number of healthy participants with 0 DALYs at the end of follow-up the outcome was not normally distributed, i.e. a peak at 0 and a normal distribution in participants with DALYs>0. Therefore, we used a two-part model [17] to estimate the relationship between smoking status and DALYs. A two-part model combines the probability of DALYs estimated using logistic regression with the number of DALYs per smoking category among participants with DALYs estimated using linear regression. Confidence intervals were constructed with bootstrapping. The relationships were adjusted for age, sex, BMI, physical activity, education, ethanol and energy intake (all measured at recruitment).

We conducted additional analyses stratifying for sex and two age categories (age at cohort entrance: <50, ≥50). To evaluate the effect of extending follow-up time we conducted additional analyses to calculate DALYs for different follow-up periods, until 2001 (average 6.4 years of follow-up), 2003 (average of 8.4 years) and 2005 (average of 10.4 years). Furthermore, DALYs were computed for specific diseases to assess which disease causes the highest attributable disease burden. For these comparisons we chose to use method 4 to estimate the life expectancy, because we believe this method is the most realistic one in the sense that it measures health losses as they actually occur.

All statistical analyses were conducted using SAS 9.2 (SAS Institute, Cary, US).

## Results

The mean follow-up of the 33,507 EPIC-NL participants was 12.4 years, 6,741 participants were identified with a non-fatal disease of interest and 1,504 died during this follow-up period. The total disease burden for the entire follow-up period was 69,245 DALYs; 40,861 (59%) healthy years were lost due to disability (YLD) and 28,384 (41%) years were lost due to premature death (YLL) (table 1). Cancer (26,363 DALYs), Coronary Heart Disease (12,896 DALYs) and Osteoarthritis (7,491 DALYs) caused most disease burden.

**Table 1:** Years Lost due to Disease (YLD), Years of Life Lost (YLL) and Disability-Adjusted Life Years (DALYs) of all EPIC-NL participants (N = 33,507).

Disease^a^	Incidences^b^	YD		DW	YLD( = YD*DW)		Deaths^c^	YLL		DALY( = YLD+YLL)		
	N	Sum	Mean		Sum	Mean	N	Sum	Mean	N^d^	Sum	Mean
CHD	1,490	32,541	21.84	0.29	9,437	6.33	184	3,459	18.80	1,626	12,896	7.93
CVA	459	8,625	18.79	0.61	5,261	11.46	108	1,833	16.97	522	7,094	13.59
Diabetes Mellitus	762	17,668	23.19	0.20	3,534	4.64	40	666	16.65	790	4,199	5.32
Cancer^e^	2,519	41,282	16.39	0.08–0.59	11,142	4.42	790	15,220	19.27	2,555	26,363	10.32
COPD	423	8,291	19.60	0.31	2,570	6.08	55	884	16.07	448	3,454	7.71
Asthma	102	2,815	27.60	0.08	225	2.21	1	29	29.00	103	254	2.47
Parkinson’s disease	42	653	15.55	0.68	444	10.57	11	131	11.91	48	575	11.98
Rheumatoid arthritis	130	3,042	23.40	0.53	1,612	12.40	1	18	18.00	131	1,631	12.45
Osteoarthritis	1,867	39,362	21.08	0.19	7,479	4.01	1	12	12.00	1,868	7,491	4.01
IBD	46	1,468	31.91	0.20	294	6.39	2	40	20.00	48	334	6.96
Other			–	–		–	380	7,242	19.06	380	7,242	19.06
Combined (method 4)	6,741	137,660	20.42	0.08–0.68	40,861	6.06	1,504	28,384	18.87	7,178	69,245	9.65
**Other methods**												
Combined (method 1)	6,741	63,775	9.46	0.08–0.68	18,565	2.75	1,504	11,746	7.81	6,295	30,311	4.82
Combined (method 2)	6,741	114,536	16.99	0.08–0.68	33,765	5.01	1,504	24,085	16.01	7,172	57,851	8.06
Combined (method 3)	6,741	133,908	19.86	0.08–0.68	39,716	5.89	1,504	28,553	18.98	7,178	68,269	9.51

Abbreviations: YD, Years with Disability; DW, Disability Weight; YLD, Years Lost due to Disease; YLL, Years of Life Lost; CHD, Coronary Heart Disease; CVA, Cerebrovascular Accident; COPD, Chronic Obstructive Pulmonary Disease; IBD, Inflammatory Bowel Disease.

a Years lost per disease presented only using life expectancy at time of death or if alive at end of follow-up (method 4), total DALYs are shown for the other life expectancy methods.

b Non-fatal incidence cases.

c Primary or secondary cause of death due to disease.

d Participants with DALYs.

e DALYs have been calculated for each specific cancer type but were aggregated for this table.

Table 2 shows the characteristics for smokers (2.60 DALYs), former-smokers (1.98 DALYs) and never-smokers (1.71 DALYs) of the EPIC-NL cohort. After multivariable adjustment former-smokers on average have 0.22 DALY (regression coefficient) (95%CI: 0.10, 0.34) and current-smokers 1.28 DALYs (95%CI: 1.10, 1.46) equaling a mean loss of 1 year and 3 months of healthy life for smokers (during 12 years in people on average 49 years old when reporting smoking status) compared to never-smokers (table 3, method 4). The association of current smoking with health was somewhat smaller when life expectancy was estimated according to the methods 1–3∶0.66 (95%CI: 0.56, 0.76) for method 1; 1.11 (95%CI: 0.96, 1.27) for method 2; and 1.25 (95%CI: 1.08, 1.43) for method 3.

**Table 2:** Characteristics of 33,507 EPIC-NL participants by smoking status^a^.

	Smoking categories		
	Never	Former	Current
N	12,908	10,403	10,196
Follow-up, years, mean (SD)	12.4 (1.6)	12.4 (1.5)	12.4 (1.6)
Age at recruitment, mean (SD)	48.9 (13.1)	50.9 (10.1)	46.4 (11.6)
Sex, % male	21.2	25.6	32.5
BMI, mean (SD)	25.7 (4.1)	25.9 (3.9)	25.1 (3.8)
**Participants, % (N)**			
Participants without a disease	80.4 (10,384)	77.5 (8,062)	77.3 (7,882)
Participants with a disease	18.5 (2,385)	21.4 (2,222)	20.9 (2,134)
Participants deceased	3.6 (463)	4.0 (421)	6.1 (620)
**Years lost, mean (total)**			
YLD	1.07 (13,874)	1.25 (13,042)	1.37 (13,945)
YLL	0.64 (8,232)	0.73 (7,588)	1.23 (12,564)
DALY	1.71 (22,106)	1.98 (20,630)	2.60 (26,509)
**Diseases, N** b **(DALY)**			
CHD	455 (3,326)	521 (3,730)	650 (5,840)
CVA	175 (2,262)	155 (1,967)	192 (2,866)
Diabetes Mellitus	310 (1,617)	255 (1,287)	225 (1,296)
Cancer^c^	868 (8,171)	845 (8,103)	842 (10,089)
COPD	106 (811)	107 (725)	235 (1,918)
Asthma	40 (93)	29 (95)	34 (66)
Parkinson’s disease	21 (264)	22 (258)	5 (53)
Rheumatoid arthritis	46 (576)	47 (619)	38 (435)
Osteoarthritis	803 (3,112)	643 (2,551)	422 (1,828)
IBD	13 (125)	16 (93)	19 (117)
Total	2,524 (22,106)	2,341 (20,630)	2,313 (26,509)

Abbreviations: YLD, Years Lost due to Disability; YLL, Years of Life Lost; DALY, Disability-Adjusted Life Year; CHD, Coronary Heart Disease; CVA, Cerebrovascular Accident; COPD, Chronic Obstructive Pulmonary Disease; IBD, Inflammatory Bowel Disease.

a YLD, YLL and DALYs in this table are calculated using life expectancy calculated at time of death or if alive at end of follow-up (method 4).

b Fatal and non-fatal cases.

**Table 3:** Regression coefficients and 95% CI of smoking status in relation to Disability-Adjusted Life Years (DALYs) in 33,507 EPIC-NL participants.

	Smoking categories		
	Never	Former	Current
Method 1^a^	reference	0.13 (0.06, 0.21)	0.66 (0.56, 0.76)
Method 2^a^	reference	0.19 (0.77, 0.30)	1.11 (0.96, 1.27)
Method 3^a^	reference	0.21 (0.09, 0.33)	1.25 (1.08, 1.43)
Method 4^a^	reference	0.22 (0.10, 0.34)	1.28 (1.10, 1.46)

a Model adjusted for age, sex, BMI, physical activity, education, ethanol and energy intake.

In sensitivity analyses, longer follow-up periods show larger effects for smoking: at the end of follow-up in 2001 (mean follow-up time: 6.4 years) smokers lost 8 months of healthy life (0.70 DALY) while this was 1 year and 3 months (1.28 DALYs) at the end of follow-up in 2007 (mean follow-up time: 12.4 years) (table 4). Stratified analyses showed that the number of DALYs for smokers compared to never-smokers were highest in the participants aged ≥50 years. Male smokers lost more healthy years (1.50 DALYs) than female smokers (1.12 DALYs) compared to male and female never-smokers. Compared to never-smokers current-smokers lost most healthy years due to Coronary Heart Disease, cancer, Cerebrovascular Accident, and Chronic Obstructive Pulmonary Disease, which thus drive the association of smoking status with DALYs.

**Table 4:** Sensitivity analysis for the relationship of smoking status with Disability-Adjusted Life Years (DALYs) in 33,507 EPIC-NL participants^a^.

	Smoking categories		
	Never	Former	Current
**End of follow-up** b			
31-12-2007	reference	0.22 (0.10, 0.34)	1.28 (1.10, 1.46)
31-12-2005	reference	0.15 (0.03, 0.28)	1.10 (0.92, 1.27)
31-12-2003	reference	0.11 (0.00, 0.22)	0.91 (0.75, 1.07)
31-12-2001	reference	0.12 (0.03, 0.22)	0.70 (0.57, 0.84)
**Age categories** b			
<50	reference	0.08 (−0.10, 0.28)	0.71 (0.49, 0.95)
≥50	reference	0.27 (0.09, 0.43)	1.64 (1.39, 1.91)
**Sex** b			
Male	reference	0.38 (0.14, 0.65)	1.50 (1.19, 1.89)
Female	reference	0.17 (0.04, 0.31)	1.12 (0.93, 1.32)
**Diseases** b			
CHD	reference	0.14 (0.06, 0.21)	0.61 (0.47, 0.74)
CVA	reference	0.03 (−0.03, 0.09)	0.19 (0.11, 0.30)
Diabetes mellitus	reference	0.00 (−0.04, 0.03)	0.07 (0.02, 0.11)
Cancer^c^	reference	0.12 (0.05, 0.22)	0.49 (0.38, 0.62)
COPD	reference	0.01 (−0.01, 0.04)	0.17 (0.11, 0.27)
Asthma	reference	0.00 (−0.14, 0.18)	0.00 (−0.02, 0.56)
Parkinson’s disease	reference	0.65 (−0.51, 2.17)	−1.17 (−3.14, 0.03)
Rheumatoid arthritis	reference	0.47 (−0.22, 1.26)	0.35(−0.38, 1.22)
Osteoarthritis	reference	−0.00 (−0.02, 0.02)	−0.02 (−0.04, 0.01)
IBD	reference	0.46 (−0.14, 1.12)	0.65 (−0.00, 1.56)

Abbreviations: CHD, Coronary Heart Disease; CVA, Cerebrovascular Accident; COPD, Chronic.

Obstructive Pulmonary Disease; IBD, Inflammatory Bowel Disease.

a DALYs in this table are calculated using life expectancy calculated at time of death or if alive at end of follow-up (method 4).

b Models adjusted for age, sex and BMI, physical activity, education, ethanol and energy intake.

c DALYs have been calculated for each specific cancer type but were aggregated for this table.

## Discussion

This paper presents several methodological issues related to the computation of DALYs in an on-going follow-up study. We show that the method of life expectancy calculation i.e. based on which age and which calendar year, drive the DALY estimates. In addition, length of follow-up is another important factor in the calculation of the total disease burden, with longer follow-up (i.e. larger extinction of the cohort) leading to larger effect sizes. We observed that during a period of 12 years -in an on average 49 years old population- smokers lost 1.28 healthy years of their life (1.28 DALYs) compared to never-smokers.

We compared four different methods to calculate life expectancy. The methods resulted in different estimations for the absolute number of DALYs, but an association between smoking status and DALYs was clear for all methods.

Method 1 in which age is constant (0) and the reference calendar year is variable (birth year) has the disadvantage that people who die at the same age from the same disease and who are equally exposed to the same risk factor but who are born in different years are assigned a different number of years lost. Consequently, the effect assigned to the risk factor depends on the birth years of the participants in a cohort. For example, in a young cohort (born later) the impact of risk factors increase. Persons who live beyond their life expectancy (as well as with method 2) obtain zero DALYs for the period after their life expectancy. The period someone lives beyond the life expectancy could also be seen as health gain (negative DALYs). When including negative DALYs in the analysis this only slightly changes the results for the association with smoking status (data not shown). Method 2, in which age and reference calendar year are constant, has a constant but arbitrary life expectancy for all individuals, possibly stratified by sex. The advantage is that persons who die at the same age lose the same number of life years and studies can be compared based on the same endpoint. However it is hard to imagine that researchers would chose an arbitrary life expectancy which has no plausible link to the participants in their cohort.

Method 3 in which age is variable (age at death) and the reference calendar year is constant (1995) has the disadvantage that the reference year is arbitrary. Furthermore, the effect of a risk factor is underestimated. Consider two people born in the same year but one dying early due to being exposed to a risk factor. The person dying later could lose almost as much life years (YLL) because relative life expectancies increase with age.

Method 4 in which both age (age of death) and reference year (year of death) are variable has the disadvantages of both method 1 and 3 but it is the most accurate in the amount of life years a person actually loses when he dies.

We propose to calculate the remaining life expectancy at time of death or when alive, at end of follow-up (method 4) because it is the most realistic method in the sense that the years of life lost for an individual are the best estimate at the time of his death or end of follow-up. Moreover, the differences between the methods turned out small, so it seems not to matter too much which method is used.

The number of DALYs for smokers is lower than several estimates previously reported [18], [19]. Several issues should be taken into consideration. First, not all relevant diseases were incorporated in our analysis, and for other major diseases only severe cases resulting in hospitalization were included. In addition, the registered date of onset of disease (date of hospital discharge) is likely to be later than the true date of onset when the disease started to contribute to disease burden. However, we did include those diseases that are most strongly associated with smoking.

Another important point is that incidence data are not complete due to the truncated nature of the ongoing cohort study. For a complete view of any effect the ideal cohort for these calculations would be the follow-up of a birth cohort until extinction. In such a study population, DALYs before baseline and after the end of follow-up are directly observed. We did not have access to an extinct birth cohort, so we had to make assumptions with regard to the YLD and YLL calculation of participants still alive at the end of follow-up. YLL for subjects still alive at end of follow-up was set at zero, assuming subjects all live until the estimated date of their life expectancy. If they were healthy at the end of follow-up they are treated as remaining healthy until their expected age of death. However, these people likely will also develop a disease before dying, so underestimating numbers of DALYs. For those participants who got a disease during follow-up (before December 2007) the YLD was calculated assuming they reach the estimated life expectancy, i.e. a date after 2007. Unfortunately there were no life tables available for specific patient groups therefore we used the same life table for all participants. In reality, participants alive at the end of follow-up may die before reaching their expected time of death, and those with a disability are more likely to die earlier, but since these premature deaths are not yet observed they were not included in the calculation. Presumably, as demonstrated in our sensitivity analysis, longer follow-up will increase the number of DALYs. The longer the follow-up period, ideally until extinction of the cohort, the greater the accuracy of the calculated association between smoking status and DALYs. The current reported loss for smokers ignores loss before study entry, refers to an observation period of only 12.4 years, underestimates DALYs due to hospital discharge dates instead of earlier incidence dates, and assumes for persons alive at end of follow-up, with or without disease, that they will live until estimated life expectancy.

We excluded participants with prevalent disease possibly related to smoking behavior at baseline. Altogether, in this relatively young and healthy cohort most participants survived more than 12 years of follow-up. Consequently, smokers who were still healthy after 12.4 years have as much DALYs as healthy never-smokers who were still healthy at the end of follow-up whereas the smokers probably will develop more disease later on and die earlier after truncation.

In conclusion, we present a methodology to calculate DALYs from real-life data. This summary outcome measure can be used to assess the prospective relationship between a determinant such as smoking status and disease burden in a cohort study. DALYs have the advantage that risk factors with small effects on many diseases can be more easily identified and the overall effect of risk factors that may have opposing impacts on different diseases can be estimated. The outcome is sensitive to two factors, the assumption used for computing the life expectancy and the follow-up time that determines the number of deaths and survivors at the end of this time. The longer the follow-up, the completer the outcome picture approaches the ultimate outcome. We believe that the use of DALYs in a prospective cohort is an appropriate way to explore the association between different lifestyle determinants and total disease burden.

## Supporting Information

Table S1 **Disability weights for different cancer types.**
(DOCX)

EPIC-NL description S1 (DOCX)
