# Supplementary material for: Development of Methodology for Disability-Adjusted Life Years (DALYs) Calculation Based on Real-Life Data
Source: PLoS One. 2013 Sep 20;8(9):e74294. doi: 10.1371/journal.pone.0074294 (PMC3779209; doi:10.1371/journal.pone.0074294)
Supplement: Table S1 — Disability weights for different cancer types. (DOCX) [file pone.0074294.s001.docx]

**Table S1 Disability weights for different cancer types**

| **Cancer type** | **Disability weight** | **Source** |
| --- | --- | --- |
| **Digestive** |  |  |
| Stomach | 0.59 | Dutch disability weight |
| Oesophagus | 0.53 | Dutch disability weight |
| Colon | 0.30 | Dutch disability weight |
| Pancreas | 0.53 | Based on dutch disability weight for oesophagus cancer |
| Unspecified parts of billary tract | 0.53 | Based on dutch disability weight for oesophagus cancer |
| Galbladder | 0.53 | Based on dutch disability weight for oesophagus cancer |
| Liver and intrahepatic bile ducts | 0.53 | Based on dutch disability weight for oesophagus cancer |
| Rectum | 0.30 | Based on dutch disability weight for colon cancer |
| Rectosigmoid junction | 0.30 | Based on dutch disability weight for colon cancer |
| Small intestine | 0.30 | Based on dutch disability weight for colon cancer |
| Anus and anal canal | 0.30 | Based on dutch disability weight for colon cancer |
| Other and ill-defined digestive organs | 0.30 | Based on dutch disability weight for colon cancer |
| **Respiratory, intrathoracic** |  |  |
| Bronchus and lung | 0.54 | Dutch disability weight |
| Heart, mediastinum and pleura | 0.54 | Based on dutch disability weight for lung cancer |
| Accessory sinuses | 0.54 | Based on dutch disability weight for lung cancer |
| Larynx | 0.54 | Based on dutch disability weight for lung cancer |
| **Male genital organs** |  |  |
| Prostate | 0.26 | Dutch disability weight |
| **Breast** |  |  |
| Breast | 0.26 | Dutch disability weight |
| **Hematopoetic and reticuloendothelial** |  |  |
| Hematopoetic and reticuloendothelial | 0.24 | Dutch disability weight |
| **Female genital organs** |  |  |
| Cervix uteri | 0.12 | Dutch disability weight |
| Vulva | 0.12 | Based on dutch disability weight for cervix uteri cancer |
| Ovary | 0.30 | Based on dutch disability weight for colon cancer |
| Corpus uteri | 0.12 | Based on dutch disability weight for cervix uteri cancer |
| Unspecified female genital organs | 0.30 | Based on dutch disability weight for colon cancer |
| **Skin** |  |  |
| Skin | 0.08 | Dutch disability weight |
| **Brain, central nervous system** |  |  |
| Brain | 0.54 | Based on australian disability weight for brain cancer |
| Spinal cord, cranial nerves, other parts of central nervous system | 0.54 | Based on australian disability weight for brain cancer |
| **Urinary tract** |  |  |
| Kidney, except renal pelvis | 0.26 | Based on dutch disability weight for prostate cancer |
| Bladder | 0.26 | Based on dutch disability weight for prostate cancer |
| Ureter | 0.26 | Based on dutch disability weight for prostate cancer |
| **Mesothelial, soft tissue** |  |  |
| Retroperitoneum and peritoneum | 0.30 | Based on dutch disability weight for colon cancer |
| Other connective and soft tissue | 0.30 | Based on dutch disability weight for colon cancer |
| **Thyroid endocrine gland** |  |  |
| Thyroid gland | 0.20 | Based on dutch disability weight for thyroid cancer |
| **Lip, oral cavity, pharynx** |  |  |
| Pyriform sinus | 0.53 | Based on dutch disability weight for oesophagus cancer |
| Tonsil | 0.53 | Based on dutch disability weight for oesophagus cancer |
| Base of tongue | 0.53 | Based on dutch disability weight for oesophagus cancer |
| Floor of mouth | 0.53 | Based on dutch disability weight for oesophagus cancer |
| Oropharynx | 0.53 | Based on dutch disability weight for oesophagus cancer |
| Nasopharynx | 0.53 | Based on dutch disability weight for oesophagus cancer |
| Gum | 0.53 | Based on dutch disability weight for oesophagus cancer |
| Other unspecified parts of tongue | 0.53 | Based on dutch disability weight for oesophagus cancer |
| Other and ill-defined sites in lip, oral cavity and pharynx | 0.53 | Based on dutch disability weight for oesophagus cancer |
| **Bone and articular cartilage** |  |  |
| Bone and articular cartilage of limbs | 0.30 | Based on australian disability weight for bone cancer |
