# Supplementary material for: Development of Methodology for Disability-Adjusted Life Years (DALYs) Calculation Based on Real-Life Data
Source: PLoS One. 2013 Sep 20;8(9):e74294. doi: 10.1371/journal.pone.0074294 (PMC3779209; doi:10.1371/journal.pone.0074294)
Supplement: EPIC-NL description S1 — (DOCX) [file pone.0074294.s002.docx]

**EPIC-NL description S1**

EPIC-NL

Disability-Adjusted Life Years (DALYs) were computed among participants of the Dutch part of the European Prospective Investigation into Cancer and Nutrition (EPIC-NL) study [1]. EPIC-NL is a prospective cohort study among men and women aged 20-70 years at entrance. Between 1993 and 1997, 40,011 participants were recruited from the general population and from women attending the National breast cancer screening. All participants provided informed consent before study inclusion. The study complies with the Declaration of Helsinki and was approved by the institutional board of the University Medical Center Utrecht (Prospect) and the Medical Ethical Committee of TNO Nutrition and Food Research (MORGEN).

In the present study participants who did not give consent to linkage with the registries (n=2,879) were excluded together with participants who suffered from any of the studied disabilities at baseline (n=3,625). This left 33,507 healthy participants for analysis.

Endpoint assessment

Participants were followed for mortality and morbidity through linkage with several registries. Information on vital status and the date of death was obtained through linkage with municipal registries. The cause of death was obtained from Statistics Netherlands. Information on disease occurrence (cancer, Coronary Heart Disease, Cerebrovascular Accident, Diabetes Mellitus, Chronic Obstructive Pulmonary Disease, Asthma, Parkinson’s disease, Rheumatoid Arthritis, Osteoarthritis, and Inflammatory Bowel Disease) was obtained from the National Cancer Registry, the national hospital discharge diagnosis database from the Dutch National Medical Registry and self-report. The National Cancer Registry provided information on the type of cancer and the date of diagnosis. The national hospital discharge diagnosis database provided the date of diagnosis for Coronary Heart Disease, Cerebrovascular Accident, Diabetes Mellitus, Chronic Obstructive Pulmonary Disease, Asthma, Parkinson’s disease, Rheumatoid Arthritis, Osteoarthritis, and Inflammatory Bowel Disease. First diagnosis of the disease was assumed to be the date of hospital discharge. The national hospital discharge diagnosis database was linked to the cohort on the following information: date of birth, gender, postal code and name of the general practitioner with a validated probabilistic method [2]. Self report and urinary glucose strip tests provided additional information on Diabetes Mellitus at study entry. New Diabetes Mellitus cases ascertained during follow up were verified using information of the general practitioner or pharmacist [3]. The selection of the diseases mentioned above was based on their prevalence and disease burden in the Netherlands, but also on the availability of data information sources. Follow-up was complete until 31 December 2007.

Exposure to risk factors and assessment of confounding factors

Information on smoking status (categorized into current-, former-, and never-smoker), education level (categorized into low (primary education up to and including those completing advanced elementary education), middle (intermediate vocational education and higher general secondary education) or high (higher vocational education and university)) was obtained from a general questionnaire filled out by all participants at baseline. The food frequency questionnaire provided information on energy intake and ethanol intake. Physical activity was assessed using the EPIC physical activity questionnaire and categorized according to the Cambridge Physical Activity Index (inactive, moderately inactive, moderately active and active) [4]. In addition, a physical examination was performed at baseline including height and weight measurement from which the BMI could be calculated (kg/m2). As information on smoking status was missing in 0.5% and physical activity in 14% of the participants we imputed these values using single linear regression modeling (SPSS, Missing Value Analysis procedure).

References

1. Beulens JWJ, Monninkhof EM, Verschuren WM, van der Schouw YT, Smit J, *et al.* (2010) Cohort profile: the EPIC-NL study. Int J Epidemiol 39: 1170-1178.
2. Herings RM, Bakker A, Stricker BH, Nap G. (1992) Pharmaco-morbidity linkage: a feasibility study comparing morbidity in two pharmacy based exposure cohorts. *J Epidemiol Community Health* 46: 136-140.
3. Sluijs I, van der A DL, Beulens JW, Spijkerman AM, Ros MM, et al. (2010) Ascertainment and verification of diabetes in the EPIC-NL Study. *Neth J Med* 68: 333–339.
4. Interact Consortium, Validity of a short questionnaire to assess physical activity in 10 European countries. (2012) Eur J Epidemiol 27: 15-25.
